# Effects of Internet Language Related to COVID-19 on Mental Health in College Students: The Mediating Effect of Cognitive Flexibility

**DOI:** 10.3389/fpsyg.2021.600268

**Published:** 2021-06-14

**Authors:** Xingzhe Wu, Zhi Wang, Hongpo Zhang, Peiming Yuan, Quanlei Yu, Zhijin Zhou, Qingbai Zhao

**Affiliations:** ^1^School of Psychology, Central China Normal University, Wuhan, China; ^2^Mental Health Education and Counseling Center, Youjiang Medical University for Nationalities, Guangxi, China; ^3^Key Laboratory of Adolescent Cyberpsychology and Behavior (CCNU), Ministry of Education, Wuhan, China

**Keywords:** Internet language, cognitive flexibility, anxiety, depression, COVID-19

## Abstract

During the COVID-19 pandemic, Internet language (INL) has influenced daily life extensively. However, the process by which INL influences people’s psychology and behavior is unclear. This study explored the effects of INL on mental health (anxiety and depression). A pilot study was conducted to develop a qualified scale for INL related to COVID-19 (CINL) in college students using an online questionnaire. The CINL scale was found to have two dimensions: frequency and comprehension, as well as good reliability and validity. A formal study explored the mediating effect of cognitive flexibility on the relationship between CINL and mental health. The results showed that CINL positively predicted mental health when it was mediated by cognitive flexibility. These results not only provide a new perspective on understanding the effects of cyber behavior on human mental health from a positive perspective, but also provide practitioners with new insights for interventions on college students’ mental health.

## Introduction

At the beginning of 2020, COVID-19 broke out worldwide, and as of April 18, 2021, the cumulative number of confirmed cases and deaths globally had reached 140,578,995 and 3,010,453, respectively (World Health Organization, 2021). COVID-19 has spread rapidly across the world and is a source of ongoing concern. It is worth noting that stress from the risks associated with the COVID-19 pandemic – social alienation, the implementation of isolation measures, and the consequences of the disease – significantly impact people’s mental health and increase the risk of mental health problems (Vahia et al., 2020). People who are diagnosed with or suspected of being infected with COVID-19 experience negative emotional and behavioral reactions, such as fear, loneliness, anxiety, and insomnia (Ornell et al., 2020). In addition, COVID-19 also has a significant negative impact on people who are not infected. A recent study conducted in China demonstrated that about 53.8% of the respondents began to suffer from a moderate or severe psychological disorder during the pandemic. In particular, 16.5% reported experiencing moderate to severe depressive symptoms, 28.8% reported experiencing moderate to severe anxiety symptoms, and 8.1% experienced moderate to severe stress (Wang et al., 2020).

A vast body of Internet language (INL) has emerged in the context of the COVID-19 pandemic. INL in China refers to a language generated or applied in online communication and is composed of Chinese and English letters, punctuation marks, symbols, pictures, characters, and so on (Wu, 2004; Du, 2007). Generally, these combinations express special meanings in online communication. Previous studies suggest that Chinese INL has many types, including those that parse Chinese characters (e.g., combination of two Chinese characters “弓” meaning “bow,” and “虽” meaning “though” into a new Chinese character “强,” meaning “strong”), stylized numbers (e.g., the number 94 sounds like “jiu shi” in Mandarin, which means confirmation), and so on. With the rapid development of the Internet, the forms and content of INL are becoming increasingly diversified. In the context of COVID-19, some common words have been given new usages or interpretations. For example, in the past, the idiom “du (度) ri (日) ru (如) nian (年),” which means “getting through a day is like getting through a year,” is used to describe the experience of going through a difficult time; however, netizens have given this idiom a new meaning: “during the pandemic, individuals do not need to work, and they can relax as they would on the lunar New Year holiday.” Another phrase that has emerged is: “before the pandemic, we were afraid to encounter ghosts; however, during the pandemic, we are afraid to encounter people.” However, it is not sufficient to merely summarize all the types of INL. Many studies have demonstrated that individuals who browse or use INL experience positive emotions that are often accompanied by insights (Ma et al., 2012; Shen et al., 2017). Therefore, INL is generally characterized as being humorous. Furthermore, understanding INL requires users to adopt unusual perspectives to interpret INL’s novel meanings (Zhou, 2010; Xu, 2015); thus, novelty is another characteristic of INL.

Generally, INL is updated quickly based on current events. Whether this refers to events in daily life or during the COVID-19 pandemic, INL has been found to influence study, work, and life. Among these, mental health, such as anxiety and depression, is also affected. On the one hand, because INL is usually humorous in tone, it can trigger positive emotions (happy, relaxed, etc.) that can promote mental health (Guo and Wang, 2007; Zhao et al., 2010). In addition, the humor of INL has an entertainment function, so the imitation and use of INL can easily close interpersonal distance, increase interpersonal attraction, and further satisfy the need for belonging (Yao and Zhou, 2019), which could also impact mental health. Therefore, we hypothesized that INL can directly improve the mental health of netizens during COVID-19.

On the other hand, because of the novelty of INL, which can help individuals to view things from multiple perspectives and improve their understanding of the same thing, INL could also improve individuals’ cognitive flexibility, which is defined as the ability of human beings to change cognitive processing strategies in response to new and unexpected situations in their environment (Canas et al., 2003; Al-Mosaiwi and Johnstone, 2018). The effect of novel stimuli on cognitive flexibility has been supported by many previous empirical studies (Barceló et al., 2002; Jowkar-Baniani and Schmuckler, 2014).

Previous studies have found that individuals with high cognitive flexibility are more likely to consider a situation from a different point of view and switch their ideas smoothly, resulting in individuals with high cognitive flexibility and higher levels of life satisfaction, self-esteem, and mental health (Kim and Omizo, 2005; Koesten et al., 2009; Windle, 2010; Jen et al., 2018; Odacı and Çikrikci, 2018). Furthermore, individuals with high cognitive flexibility are more likely to meet the requirements of the environment, efficiently cope with difficult situations, adapt their thoughts and behaviors, and be good at problem solving. This leads to a higher likelihood of success in settling individual internal conflicts and clashes with the outside world, and in establishing a good balance with the outside world (Tchanturia et al., 2004; Hasselbalch et al., 2012; Murphy et al., 2012; Brewster et al., 2013; Mehri and Bakhtiarpoor, 2016; Perpiñá et al., 2016; Jahja et al., 2017; Edwards, 2019). Therefore, this study further hypothesized that INL may have an indirect effect on mental health through cognitive flexibility during COVID-19.

In other words, while people are at risk of mental health issues during the pandemic, INL may affect the mental health of netizens through direct and indirect paths. Therefore, this study synthetically assumed that INL promotes netizens’ mental health during COVID-19, which may be partially mediated by cognitive flexibility.

To explore the effect of INL on mental health during the COVID-19 pandemic, a pilot study was conducted to develop a scale for INL in relation to COVID-19 (CINL). A cross-sectional survey was conducted to explore the mediating effect of cognitive flexibility on the relationship between CINL and mental health. To obtain sufficient and suitable data, this study developed an online questionnaire for data collection. Previous studies have found that mental health is influenced by participants’ gender (Zhang and Yu, 2004; Feng and Zhang, 2005; He, 2014), age (Zhang et al., 2011), physical health status (Dong et al., 2012; Lu and Wang, 2015), family economic status (Hudson, 2005; Hu et al., 2014), and physical activity (Swann et al., 2018; Zheng et al., 2020). To eliminate the interference of these variables, this study included these variables as control variables.

## Pilot Study: Developing a Scale of Inl Related to COVID-19

Although previous studies have focused on the use of INL (Liu et al., 2017, 2019; Zhao et al., 2017; Zhang et al., 2019), there are no qualified tools to measure INL. Furthermore, a scale for INL related to COVID-19 requires specificity and effectiveness. Therefore, to explore the effect of CINL on mental health, it was necessary to develop a scale for CINL with good reliability and validity.

### Theoretical Construction

As suggested by previous studies (Yang et al., 2006; Yang and Li, 2007; Yu et al., 2017), frequency are often used as indicators to measure behavior. This study adopted *frequency* as one dimension of CINL. As the humorous or pleasant emotions aroused by INL are usually transient rather than lasting, people may need to use INL over sufficient duration and frequency to maintain a good state of mind to eliminate anxiety and depression. Furthermore, INL is different from other types of Internet communication; it is not only a behavior but also a cognitive process. The effect of INL may also depend on how it is understood (Huang et al., 2013). Take the idiom “du (度) ri (日) ru (如) nian (年)” as an example. If netizens understood this idiom in its original rather than its novel meaning, the INL would have no effect. It is remarkable that novelty is relative but not permanent; it may have been amazing for people to travel by plane, but now it is normal to fly around the world. Therefore, this study considers *comprehension* as another dimension of INL.

Based on this, the research team, which included four college teachers and three psychology graduates, developed a scale that included items in two dimensions. The frequency dimension consisted of three items (e.g., “The number of times I browse words, sentences, or paragraphs with INL related to COVID-19”) and the comprehension dimension consisted of four items (e.g., “In my life, I will try my best to use these words, sentences, or paragraphs with INL related to COVID-19”). This pilot study further established the validity and reliability of the CINL scale.

### Structural Validation

#### Participant

In total, 526 university students were recruited from a medical college in Guangxi Zhuang Autonomous Region (GZAR), China. To eliminate the possible effects of confounding variables, 116 participants were excluded because they had provided very similar responses in the survey. Finally, valid data from 416 participants were obtained (79.09% of the original sample size). The sample (*N* = 416) was randomly divided into two subgroups. Data from subgroup 1 were subjected to an exploratory factor analysis; it included data from 219 participants whose ages ranged from 18 to 23 years old, with an average age of 19.76 years (*SD* = 1.19), of whom 84 were male (38.40%). Data from subgroup 2 were subjected to a confirmatory factor analysis, and this subgroup included 197 respondents whose ages ranged from 17 to 31 years old, with an average age of 19.91 years (*SD* = 1.56), of whom 87 were male students (44.20%).

#### Validity and Reliability Analysis

##### Discrimination

An item–total correlation was used to test for discrimination of items, as suggested by classical test theory (CTT) (Lord and Novick, 1968). When the correlation coefficient between the item score and total score was greater than 0.40, the item was retained. As shown in Table 1, all the item–total correlation coefficients ranged between 0.43 and 0.73. Therefore, no items were excluded from this study.

**TABLE 1:** Correlation coefficient between each item score and total score.

Item	1	2	3	4	5	6	7
Score	0.69	0.72	0.72	0.61	0.57	0.43	0.57

##### Exploratory factor analysis (EFA)

Data from subgroup 1 were subjected to exploratory factor analyses. First, Kaiser-Meyer-Olkin (KMO) and Bartlett sphere tests were conducted, and the results indicated that the data were suitable for factor analysis (KMO = 0.74; Bartlett Sphere: χ^2^ = 573.54, *p* < 0.001). Subsequently, the principal component analysis method was used to extract factors with an eigenvalue greater than 1, and the maximum variance method was used to rotate the component matrix. Two factors were extracted, and the total contribution rate was 67.64%. According to CTT (Lord and Novick, 1968), items should not load two or more factors, and its factor load should not be less than 0.3; otherwise, the item should be dropped. As shown in Table 2, the sixth item loaded two factors and was excluded. Finally, the remaining six items were retained. The exploratory factor analysis was performed again with six items, as shown in Table 3. After factor rotation, these six items could load on two factors, which explained 73.42% of the total variation rate. These two factors were consistent with the theoretical construction; thus, this study named the two factors “frequency of use of CINL” and “comprehension of use of CINL.”

**TABLE 2:** Results of EFA of the CINL scale before revision.

	Factor load	
	1	2
1. How many time I browse words, sentences or paragraphs using INL about COVID-19	0.88	0.02
2. How much time I spend in browsing words, sentences or paragraphs using INL about COVID-19	0.88	0.07
3. How frequently I browse words, sentences or paragraphs using INL about COVID-19	0.89	0.12
4. I feel happy when I browse words, sentences or paragraphs using INL about COVID-19	0.01	0.88
5. I feel engaged when I browse words, sentences or paragraphs using INL about COVID-19	−0.05	0.86
6. I feel impressed when I browse words, sentences or paragraphs using INL about COVID-19	0.38	0.54
7. In daily life, I will try my best to use INL words, sentences or paragraphs about COVID-19	0.14	0.70

**TABLE 3:** Results of EFA of the CINL Scale after revision.

	Factor 1		Factor 2	
	Item	Loading	Item	Loading
	1	0.88	4	0.86
	2	0.89	5	0.87
	3	0.90	7	0.70
Eigenvalues	2.40	2.01		
Cumulative (%)	39.93%	73.42%		

##### Confirmatory factor analysis (CFA)

A confirmatory factor analysis was performed using data from subgroup 2. The macro program Amos was used to conduct a confirmatory factor analysis to test the two-factor model obtained by exploratory factor analysis. The results (Table 4) indicate that the two-factor model of the CINL scale has good structural validity.

**TABLE 4:** Results of confirmatory factor analysis of the CINL scale.

χ^2^	df	χ^2^/df	RMSEA	NFI	RFI	CFI	IFI
11.033	8	1.38	0.044	0.98	0.96	0.99	0.99

##### Reliability analysis

Cronbach’s alpha and split-half tests were conducted to establish the reliability of the CINL scale. Results showed that the Cronbach’s alpha of the frequency and comprehension dimensions were 0.88 and 0.74, respectively. The split-half reliability coefficients of the two dimensions were 0.86 and 0.60, respectively.

### Discussion

This pilot study developed a CINL scale that has two dimensions, namely, frequency and comprehension. The results of the validity and reliability analysis met psychometric requirements. However, the reliability of the comprehension dimension of the CINL scale was 0.60, which is relatively low. This may be because this dimension has fewer items (Angoff, 1953; Friedland and Michael, 1987; Hellman et al., 2006).

The CINL scale is the first tool to comprehensively measure behavior related to INL usage, and provides a theoretical and practical basis for future studies to explore the relationship between INL and human psychology and behaviors.

## The Mediating Effect of Cognitive Flexibility Between CINL and Mental Health

Based on the results of the pilot study, this study further explored the mediating effect of cognitive flexibility on the relationship between CINL and mental health.

### Participants

The sample was the same as in Study 1.

### Measures

#### Measurement of CINL

A self-compiled scale was used to measure CINL. Responses to the items were rated on a 5-point Likert scale, ranging from 1 (*strongly disagree*) to 5 (*totally agree*). The sum of the scores was considered an indicator of the influence of CINL. See Study 1 for detailed usage methods.

#### Cognitive Flexibility

The cognitive flexibility scale (CFI) (Dennis and Wal, 2010), which was translated into Chinese by Wang et al. (2016), was used to measure participants’ cognitive flexibility. This scale is composed of the following dimensions: controllability and choice. The controllability dimension contains 12 items (e.g., “I am good at analyzing and evaluating various situations”), while the choice dimension contains eight items (e.g., “I can always view difficulties from different angles”). The scale is rated on a 5-point Likert scale, ranging from 1 (*never*) to 5 (*always*). The total score is considered an indicator of cognitive flexibility. A higher total score indicates higher cognitive flexibility. This scale has been proven to have satisfactory reliability and validity in the Chinese context (Yang et al., 2016). In this study, Cronbach’s alpha was 0.90 and the split-half reliability coefficient was 0.87.

#### Mental Health

As suggested by previous studies (Tajima, 2001; Hoge et al., 2004; Hedelin and Jonsson, 2010; Spijker et al., 2010; Harris et al., 2015; Evans-Lacko et al., 2017), this study adopted depression and anxiety as indicators of participants’ mental health.

##### Depression

A self-rated depression scale (SDS) (Zung, 1965) was used to measure participants’ depression. This scale contains 20 items (e.g., “I feel depressed,” “I do not sleep well at night,” “I do not feel as happy as I did before when I had close contact with the opposite sex”) that are rated on a 5-point Likert scale, ranging from 1 (*strongly disagree*) to 5 (*totally agree*). Half of the items were scored in reverse. The total score is considered an indicator of depression. The higher the total score, the more severe the depression. In this study, Cronbach’s alpha was 0.86 and the split-half reliability coefficient was 0.82.

##### Anxiety

A self-rated anxiety scale (SAS) (Zung, 1971) was used to assess subjective anxiety symptoms. This scale contains 20 items (e.g., “I feel more nervous and anxious than usual,” “I feel scared for no reason,” and “I get upset or frightened easily”) that are rated on a 5-point Likert scale ranging from 1 (*strongly disagree*) to 5 (*totally agree*). Some items were scored in reverse. The total score is considered an indicator of anxiety. The higher the total score, the more severe the anxiety. In this study, Cronbach’s alpha was 0.87 and the split-half reliability coefficient was 0.79.

#### Demographic Measure

Participants’ demographic information was gathered, including their gender, age, physical health status, family economic status, and level of physical activity.

### Statistical Analyses

SPSS version 22.0 was used for the data analysis. First, a correlation analysis was used to describe the data. Second, a mediation effect test was adopted to explore the mediating effect of cognitive flexibility on the relationship between CINL and mental health, which was indicated by anxiety and depression, respectively.

### Results

#### Preliminary Analysis

As shown in Table 5, there was a significant positive correlation between CINL scores and cognitive flexibility, and a significant negative correlation between CINL and depression; however, the correlation between CINL and anxiety was not significant. Cognitive flexibility was negatively correlated with anxiety and depression. A significant positive correlation was found between anxiety and depression.

**TABLE 5:** Descriptive statistics and correlation matrix for each variable (*N* = 416).

Variable	1	2	3	4	5	6	7	8
1. Age	1							
2. Physical health status	–0.06	1						
3. Family financial status	–0.07	–0.01	1					
4. Physical activity status	0.02	0.28***	0.10*	1				
5. CINL	–0.04	–0.05	0.20***	0.14**	1			
6. Cognitive flexibility	0.05	0.19***	0.13**	0.25***	0.16**	1		
7. Anxiety	0.06	−0.22***	−0.20*	−0.13**	–0.07	−0.44***	1	
8. Depression	0.04	−0.19***	−0.22***	−0.19***	−0.17***	−0.43***	0.76***	1
*M*	19.83	1.97	2.79	2.27	17.32	66.85	48.80	50.79
*SD*	1.38	0.43	0.59	0.66	3.73	8.97	10.56	9.64

*p < 0.05, **p < 0.01, ***p < 0.001.

#### Relationship Between CINL and Mental Health: Test Model for the Mediating Effect of Cognitive Flexibility

The PROCESS of SPSS macro program model 4 was used to explore the mediating effect of cognitive flexibility on the relationship between CINL and depression, with CINL as an independent variable, cognitive flexibility as an intermediary variable, and depression as the dependent variable. Age, gender, family economic status, physical health status, and physical activity status were used as control variables.

**FIGURE 1:**
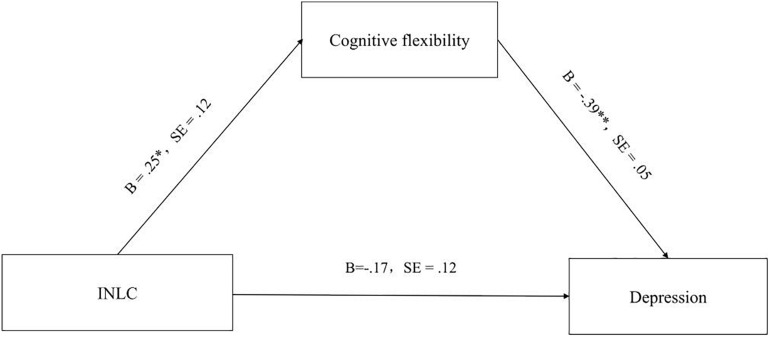
The mediating of effect of cognitive flexibility on the link between CINL and depression in the entire sample.

As shown in Figure 1, after controlling for gender, age, family economic status, physical health status, and physical activity status, CINL was found to be significantly related to cognitive flexibility, and cognitive flexibility was significantly related to depression. However, the effect of CINL on depression was not significant in this model. The Sobel test showed that the mediation model was significant (*B* = −0.10, SE = 0.006, *z* = −2.72, *p* < 0.05). The boost SE 95% confidence interval was [−0.2232, −0.0014] and did not include 0. Furthermore, the ratio of the indirect effect to the total effect was 36.93%. This suggests that cognitive flexibility fully mediates the relationship between CINL and anxiety.

Similarly, the PROCESS of SPSS macro program model 4 was used to explore the mediating effect of cognitive flexibility on the relationship between CINL and anxiety. This study used CINL as an independent variable, cognitive flexibility as an intermediary variable, and anxiety as the dependent variable, while age, gender, family economic status, physical health status, and physical activity status were set as control variables.

**FIGURE 2:**
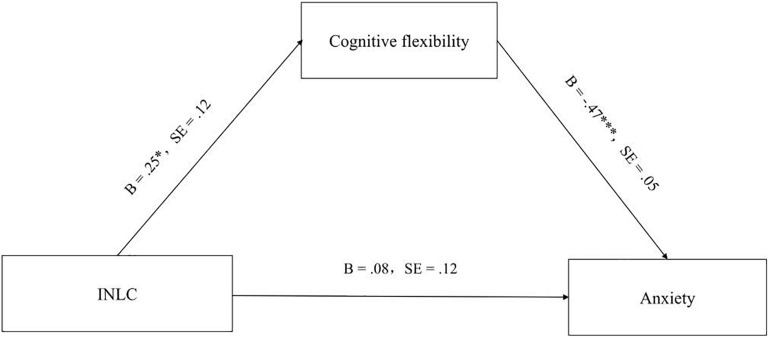
The mediating of effect of cognitive flexibility on the link between CINL and anxiety.

As shown in Figure 2, after controlling for gender, age, family economic status, physical health status, and physical activity status, CINL significantly predicted cognitive flexibility, and cognitive flexibility was a significantly negative predictor of anxiety. The Sobel test showed that the mediation model was significant (*B* = −0.12, SE = 0.006, *z* = −3.08, *p* < 0.05). The boost SE 95% confidence interval was [−0.2232, −0.0014] and did not include 0. This suggests that cognitive flexibility fully mediates the relationship between CINL and anxiety.

In a word, cognitive flexibility played a full but not partly mediating role in CINL and mental health, which partially supported the hypothesis.

## Discussion

This study explored the effects of CINL on mental health. The results showed that CINL improved mental health by promoting cognitive flexibility, which supports our hypothesis. These results are of great theoretical and practical significance in understanding the mechanisms of cyber behavior and mental health.

With regard to the demographic variables, the results of this research are partially consistent with those of previous studies on physical health status (Dong et al., 2012; Lu and Wang, 2015), family economic status (Hudson, 2005; Hu et al., 2014), and physical activity status (Swann et al., 2018; Zheng et al., 2020), with a significant positive relationship with mental health. In addition, the differences in mental health between genders were found to be marginally significant in this study, which is consistent with previous studies that have shown mental health of women was slightly lower than that of men (Zhang and Yu, 2004; Feng and Zhang, 2005; He, 2014). With regard to age, the study did not find a significant relationship between age and mental health, which differs from a previous study (Zhang et al., 2011). This may be due to the limited age range included in this study.

As expected, this study found that CINL was significantly and positively correlated with cognitive flexibility, and negatively correlated with depression. However, CINL was not significantly associated with anxiety. After controlling for cognitive flexibility, CINL was shown to have a positive effect on anxiety, although this was still not significant and somewhat different from the hypothesis. This deviation may be due to the fact that CINL may inadvertently trigger an individual’s panic and anxiety toward the COVID-19 epidemic. Previous studies have demonstrated that when facing stressors, individuals produce corresponding stress responses, such as symptoms related to an excited sympathetic nervous system, high secretion of pituitary and adrenal corticosteroids, and accelerated respiration (McGraw et al., 2013). These physiological responses are easily associated with anxiety. From this perspective, CINL’s impact on anxiety may be masked by the inhibiting effect of CINL mediated through cognitive flexibility, which may also mask the effect of exposure to CINL on anxiety. Therefore, the overall effect of CINL on anxiety was not significant.

This study hypothesized that cognitive flexibility plays a partial mediating role between CINL and mental health; however, the results showed that cognitive flexibility played a fully mediating effect. This may be because positive emotions, which are triggered by CINL, can promote individuals’ cognitive flexibility (Liang and Chen, 2015; Wang et al., 2017). Therefore, cognitive flexibility plays a full mediating role between CINL and mental health.

Previous studies on mental health have focused on cognitive factors such as cognitive flexibility, creativity, and coping strategies (Li, 2003; Luo and Lin, 2006; Wei and Fu, 2007), as well as social factors such as social support, social adaptation, and stress events (Wen et al., 2000; Ding et al., 2005; Tu and Guo, 2010). However, few studies have combined two or more of these factors to examine them simultaneously. Nowadays, the development of information is rapid; the Internet as a new medium of information communication and as an interpersonal communication tool is changing modern life and it is becoming increasingly important in people’s studies, work, life, and mental health (Chen, 2001).

Internet language lies at the core of Internet information transmission, and may have an effect as a result of a combination of factors. First, CINL is updated rapidly and is closely related to social events, requires individuals to be knowledgeable and have their own understanding of social events, and can undoubtedly increase the social adaptability of individuals. In the process of conducting the survey, we found that people with different social statuses and from demographic categories use different INLs. When the INL that an individual uses is commonly used in his/her groups, this may improve the individual’s social identity and sense of belonging, thus promoting individual social support, which is important for mental health (Pang, 2009; Kong et al., 2010). These studies suggest that INL not only plays an important role on the Internet, but also covers all aspects of real life and impacts more than just mental health. However, INL has not yet become a focus of the majority of researchers. This study preliminarily explores the relationship between INL and mental health while considering the mediating effect of cognitive flexibility, which provides a direction for future research on INL.

In addition, this study also has practical significance for interventions regarding college students’ mental health. On the one hand, we can promote the development of college students’ mental health through their preferred INL. On the other hand, due to the adverse effects of Internet use, some people, especially parents, are afraid of too much exposure to the Internet (Lei et al., 2012; Kuss et al., 2013; Lam, 2014; Yeh and Cheng, 2016; Cheung et al., 2018). They are worried that their children’s exposure to the Internet will affect their physical and mental health development. According to the results of this study, it is advisable to take an inclusive attitude toward the Internet.

This study had several limitations. First, this study used a cross-sectional paradigm; therefore, a causal relationship could not be established. Thus, future studies should adopt longitudinal cross-sectional studies or experimental methods to improve internal validity. Second, the study used a self-report method, which might not accurately indicate the use of CINL and mental health conditions. Thus, future studies should adopt more accurate indicators, such as big data analysis of CINL and objective physiological measurements of mental health. Finally, this study discusses the effect of INL on mental health in general; however, the effects of different types of INL on mental health may vary, which warrants further discussion.

## Conclusion

In the context of the COVID-19 pandemic, CINL could alleviate individuals’ depression and anxiety through the mediating pathway of cognitive flexibility. Given that most of the previous research on cyber behavior has focused on its negative effects, the results of this study provide a positive perspective on understanding the effects of cyber behavior on human mental health that may help researchers to gain a more comprehensive insight into cyber behaviors. Furthermore, INL is easy and convenient to use in daily life, so it also provides practitioners with guidance for mental health interventions.

## Data Availability Statement

The raw data supporting the conclusions of this article will be made available by the authors, without undue reservation.

## Ethics Statement

The studies involving human participants were reviewed and approved by the School of Psychology, Central China Normal University. The patients/participants provided their written informed consent to participate in this study.

## Author Contributions

ZW and XW: investigation and writing the original draft. QY, ZZ, and QZ: conceptualization, formal analysis, investigation, writing original draft, and project administration. HZ and PY: methodology, review writing, and editing. XW and QY: revised the manuscript. All authors approved the final version of the manuscript for submission.

## Conflict of Interest

The authors declare that the research was conducted in the absence of any commercial or financial relationships that could be construed as a potential conflict of interest.
